# Continuous Assessment of Function and Disability via Mobile Sensing: Real-World Data-Driven Feasibility Study

**DOI:** 10.2196/47167

**Published:** 2023-10-30

**Authors:** Emese Sükei, Lorena Romero-Medrano, Santiago de Leon-Martinez, Jesús Herrera López, Juan José Campaña-Montes, Pablo M Olmos, Enrique Baca-Garcia, Antonio Artés

**Affiliations:** 1 Department of Signal Theory and Communications Universidad Carlos III de Madrid Leganés Spain; 2 Evidence-Based Behavior S.L. Leganés Spain; 3 Kempelen Institute of Intelligent Technologies Bratislava Slovakia; 4 Faculty of Information Technology Brno University of Technology Brno Czech Republic; 5 Grupo de Tratamiento de Señal Gregorio Marañón Health Research Institute Madrid Spain; 6 Department of Psychiatry University Hospital Rey Juan Carlos Móstoles Spain; 7 Department of Psychiatry General Hospital of Villalba Madrid Spain; 8 Department of Psychiatry University Hospital Infanta Elena Madrid Spain; 9 Department of Psychiatry Madrid Autonomous University Madrid Spain; 10 Centro de Investigacion en Salud Mental Carlos III Institute of Health Madrid Spain; 11 Department of Psychiatry Universidad Catolica del Maule Madrid Spain; 12 Department of Psychiatry Centre Hospitalier Universitaire Nîmes France; 13 Department of Psychiatry University Hospital Jimenez Diaz Foundation Madrid Spain

**Keywords:** WHODAS, functional limitations, mobile sensing, passive ecological momentary assessment, predictive modeling, interpretable machine learning, machine learning, disability, clinical outcome

## Abstract

**Background:**

Functional limitations are associated with poor clinical outcomes, higher mortality, and disability rates, especially in older adults. Continuous assessment of patients’ functionality is important for clinical practice; however, traditional questionnaire-based assessment methods are very time-consuming and infrequently used. Mobile sensing offers a great range of sources that can assess function and disability daily.

**Objective:**

This work aims to prove the feasibility of an interpretable machine learning pipeline for predicting function and disability based on the World Health Organization Disability Assessment Schedule (WHODAS) 2.0 outcomes of clinical outpatients, using passively collected digital biomarkers.

**Methods:**

One-month-long behavioral time-series data consisting of physical and digital activity descriptor variables were summarized using statistical measures (minimum, maximum, mean, median, SD, and IQR), creating 64 features that were used for prediction. We then applied a sequential feature selection to each WHODAS 2.0 domain (cognition, mobility, self-care, getting along, life activities, and participation) in order to find the most descriptive features for each domain. Finally, we predicted the WHODAS 2.0 functional domain scores using linear regression using the best feature subsets. We reported the mean absolute errors and the mean absolute percentage errors over 4 folds as goodness-of-fit statistics to evaluate the model and allow for between-domain performance comparison.

**Results:**

Our machine learning–based models for predicting patients’ WHODAS functionality scores per domain achieved an average (across the 6 domains) mean absolute percentage error of 19.5%, varying between 14.86% (self-care domain) and 27.21% (life activities domain). We found that 5-19 features were sufficient for each domain, and the most relevant being the distance traveled, time spent at home, time spent walking, exercise time, and vehicle time.

**Conclusions:**

Our findings show the feasibility of using machine learning–based methods to assess functional health solely from passively sensed mobile data. The feature selection step provides a set of interpretable features for each domain, ensuring better explainability to the models’ decisions—an important aspect in clinical practice.

## Introduction

### Background

Functional limitations are associated with poor clinical outcomes, higher mortality, and disability rates, especially in older adults [1]. Moreover, they are closely related and used for predicting transitions in activities of daily living or instrumental activities of daily living due to a disability, thereby significantly impacting the quality of life of older adults and other age groups [2]. COVID-19 has been associated with functional limitations in patients with post–COVID-19 sequelae, further increasing an already present problem for older adults [3-5]. Sarcopenia, progressive muscle loss due to aging, is one of the common functionality problems for older adults with an estimated cost of hospitalizations for adults in the United States of US $40.4 billion [6]. Early detection of an increase in disability is essential for clinical practice. It can still be stabilized or even reversed in the early stages, as in the case of sarcopenia, which can be prevented, treated, and reversed by exercise. Moreover, one of the cornerstones of rehabilitation research is the reduction of disability and restoration of function [7].

There is a great need and much to be gained from defining a way to measure functioning and disability on a relevant scale, ideally daily. However, assessing everyday functioning and disability is complicated due to current measurement modalities (eg, self-report, proxy report, and clinician rating [8]). These reports are time-consuming and tedious to fill on follow-up visits. In addition, disciplines have disagreements about what constitutes a disability and the methods to measure this disability especially in a clinical setting [9]. Ecological momentary assessment (EMA) allows for more continuous assessment and monitoring of patients without face-to-face appointments and has the crucial advantage of providing data that is more relevant to daily life [10]; however, it still requires active patient input, leading to refusal and attrition. Developing adequate passive EMA tools may increase retention and help overcome the limitations of active EMA [11].

Patient-reported outcome measures (PROMs) and patient-reported experience measures (PREMs) [12] are increasingly recognized as tools providing valuable information about patients’ health statuses and perceptions of treatment at a particular time. Including such tools in the health care workflow aims to provide a patient-centered, value-based health care system [13]. A commonly used PROM for disability assessment is the second version of the World Health Organization Disability Assessment Schedule (WHODAS 2.0) [14]. This 36-item questionnaire provides a generic tool to measure health and disability. It assesses difficulties due to health conditions, including diseases or illnesses, short or long-lasting health problems, injuries, mental or emotional problems, and substance use disorders [15]. WHODAS 2.0 captures the level of functioning in six domains of life: (1) cognition—understanding and communicating; (2) mobility—moving and getting around; (3) self-care—attending to one’s hygiene, dressing, eating, and staying alone; (4) getting along—interacting with other people; (5) life activities—domestic responsibilities, leisure, work, and school; and (6) participation—joining in community activities and participating in society. Respondents are asked to reflect over the last 30 days and answer a series of questions, thinking about how much difficulty they had doing the given activities. There is a possible maximum score of 5 points for all items, indicating a rising level of difficulty in performing the activity (1: none, 2: mild, 3: moderate, 4: severe, and 5: extreme). A higher final score value, calculated as a total score or score by domain, indicates a higher level of disability [16].

Mobile sensing offers various sources, such as GPS, accelerometer, gyroscope, and light sensor that can be used to implement behavioral measures [17]. Unlike traditional assessment tools, these technologies enable long-term passive and ecological measurement of patient function. While there has been some work in digital mental health and machine learning [18], no studies predict WHODAS 2.0 functionality score changes using smartphone sensor data. These approaches are particularly important since they may enable the analysis of individuals’ functioning and disability evolution and provide a clinical tool to monitor the progression and efficacy of treatment. In addition, they provide the opportunity to build targeted, just-in-time adaptive interventions [19] in a designated population. Such frameworks deliver interventions within the context of daily life. Including passive data-driven solutions as part of the typical PROM frameworks could enrich existing information and better inform decisions.

### Goal of This Study

This work aims to provide a baseline analysis of the feasibility of using machine learning to predict patients’ WHODAS 2.0 functionality scores from passively gathered digital biomarkers. Furthermore, we aim to determine which behavioral features are the most important for predicting different WHODAS 2.0 domains. Using these features, we train a linear regressor for simplicity and interpretability of the biomarkers as predictors. This paper presents a continuous passive assessment modality and its feasibility in predicting a particular disability scale.

## Methods

### Ethical Considerations

The data used in this study were collected from an ongoing study involving passive smartphone monitoring of clinical outpatients (reference number EO 46-2013). The study received approval from the institutional review board at the Psychiatry Department of Fundación Jimenez Diaz Hospital, and all participants provided written informed consent. The institutional review board approved the secondary analysis without additional consent.

The study recruited patients who were at least 18 years old and were clinical outpatients diagnosed with mental disorders or attending therapy groups at the institutions mentioned above. To participate, patients had to own a smartphone with either Android or iOS operating systems, which they used to connect to a Wi-Fi network at least once a week. The patients did not receive payment for their participation. To protect the patient’s privacy, the study data were anonymized.

### Data Set

A clinically validated eHealth platform, eB2 Mindcare [20,21], collected the participants’ passive data. In addition, clinicians used the MEmind [22] electronic health tool to record the patients’ WHODAS 2.0 scores. Patients with the option of electronic self-reporting filled in an equivalent electronic WHODAS 2.0 self-report. Patients were able to complete the WHODAS 2.0 in parts by domain, which allowed for flexibility and less disruption to the patients. This led to later difficulties in analyzing WHODAS 2.0 total scores (officially called WHODAS 2.0 summary score computed by summing the 6 domain scores and adjusting to a range from 0 to 100) due to uncompleted domains and timing differences of when a patient would complete each domain. Thus, our predictions are focused on domain scores that allow for a larger user pool and accurate time windows for mobile data.

### Mobile-Sensed Data

The eB2 MindCare [20,21] mobile app (Evidence-Based Behavior) collects data from different sources (mobile phone sensors and wearables) at different frequencies. For this work, we focused on the data streams related to patient mobility (daily step count; distance traveled; the number of locations visited by the patient; time spent at home; and time spent performing activities such as walking, running, and exercising) and time spent asleep. Daily summaries were calculated on the values of these variables, which were then used to extract 64 descriptive statistical features for characterizing the patient’s behavior in a 30-day interval.

### Data Preprocessing

**Figure 1:**
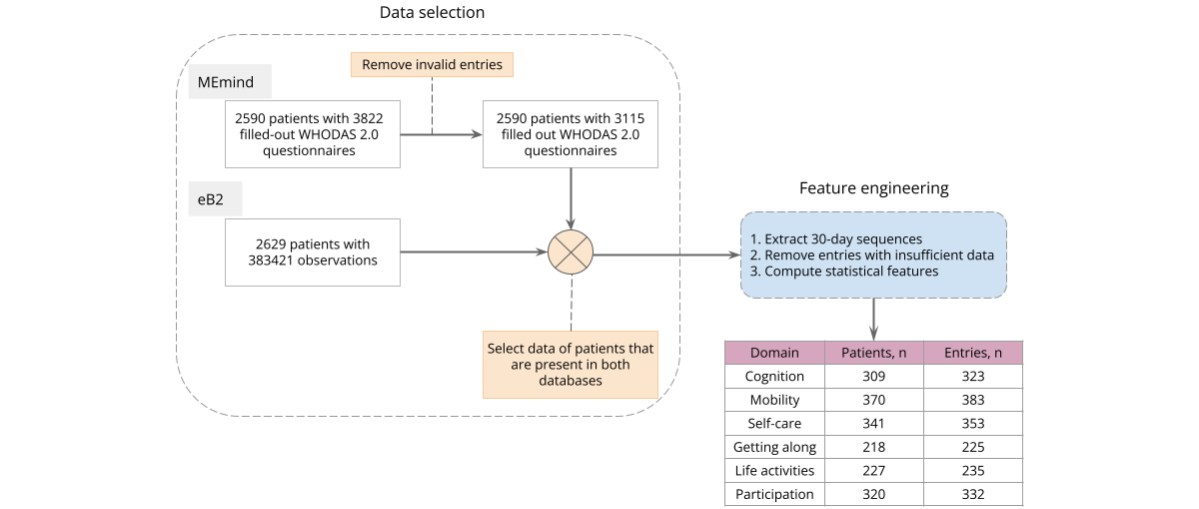
Data selection and feature engineering flowchart. WHODAS: World Health Organization Disability Assessment Schedule.

Figure 1 shows the data selection and feature extraction process. For each domain, users with the following were removed: incomplete answers (if some questions were not answered within a specific domain) and incorrect scores for the individual questions (if the registered score was out of the range of the possible scores). In addition, the particular questions’ scores from 1 to 5 were rescaled following the World Health Organization guidelines [16] to either values from 0 to 4 or 0, 1, 1, 2, 2. Finally, the score by domain was computed as the sum of the scores of the respective questions. These scores served as our target for the supervised prediction problem.

To build the input data set, we cropped a 30-day window of the data sequences for each WHODAS 2.0 entry. For the baseline WHODAS 2.0 score completed at study enrollment, we consider the next 30 days of observations because no previous mobile-sensed data were collected. For follow-up scores, which are usually collected biannually, we centered the window on encapsulating 15 days before and after the score. It was necessary to transform the time-series data set to be modeled as a nonsequential supervised learning problem. Therefore, we extracted statistical summary features (count, minimum, maximum, mean, SD, and IQR) from the sequences for each variable and obtained a data set of 64 features. We filtered the sequences for comprehensive statistics calculation by requiring every feature to contain at least 2 counts (days) of data and removing missing values.

We divided the data sets for each domain into 2 independent subsets based on the patients, ensuring no overlap. The first subset is the training data set, consisting of 80% of the entries used to fit the feature selection and predictive models. The second data set was held out for testing the model performance. The training set was split into 4 equal folds of 20% for cross-validation. The train test and cross-validation splits were done by ensuring the grouping of entries of the same user within the same set or fold since the model is user independent and stratifying the IQR of the WHODAS 2.0 scores. The stratification ensures the model can train and test low, middle, and high population WHODAS 2.0 scores. Subsequently, the features were standardized by centering the values around zero mean with a unit SD. Moving features to a similar scale helps avoid feature weight problems and provides an interpretable bias in the case of linear regression.

### Feature Selection and Predictive Modeling

We used sequential forward selection (SFS) [23], also known as a sequential feature selection or stepwise forward selection, a greedy search algorithm for feature selection, which reduces an initial *d*-dimensional feature space to a *k*-dimensional feature subspace, where *k*<*d*. SFS avoids the feature selection stability problems of the LASSO with a similar idea as best subset selection but on a reduced set of subsets, which is computationally feasible [24]. In SFS, features are added one by one to an empty set of features until reaching an upper bound set of features. At each step, a criterion or score (in our case mean absolute error [MAE]) is calculated and saved that is used to select the feature that provides the better score (lower MAE) before continuing on to the next step. To find the best set of features in the case of each domain, we performed a search by iterating from *k*=1 to 20 with 4-fold cross-validation over the training set and selecting the *k* with the highest average performance across folds with the same model design as our final model. Finding the best features for predicting each domain provides greater interpretability to our models, which is essential in a clinical setting where clinicians need reliable and straightforward decision rules [25].

Once the best features were found, we trained linear regression models to perform the prediction task. To better suit the ordinal classification problem, we performed a simple modification after the regressor by thresholding the predictions between the minimum (0) and maximum values of the specific domain by rounding. While linear regression has limitations in this specific setting, it does offer certain advantages. Linear regression models are less prone to overfitting, especially when dealing with the noise commonly encountered in real-world data. Although nonlinear models may perform better, they are more prone to overfitting and are less interpretable and explainable. These latter factors are crucial considerations when applying machine learning in a clinical setting [26,27].

The final models underwent evaluation using the held-out test set. Test MAE and test mean absolute percentage error (MAPE) served as the evaluation metrics. MAE was chosen for its linearity, which aligns well with the context of this study. This is in contrast to root-mean-squared error, which tends to overemphasize larger errors due to the squaring of error values. Given the inherent variability in real-world data sets like the passive sensing data set in this study, metrics that are more robust against larger errors were preferred [28,29]. Furthermore, MAPE provides a metric to compare the different domains with a different number of questions and different total scores. We applied this approach separately for the different WHODAS 2.0 domains, using all the extracted features and the best subset of features.

## Results

### User Statistics

**Figure 2:**
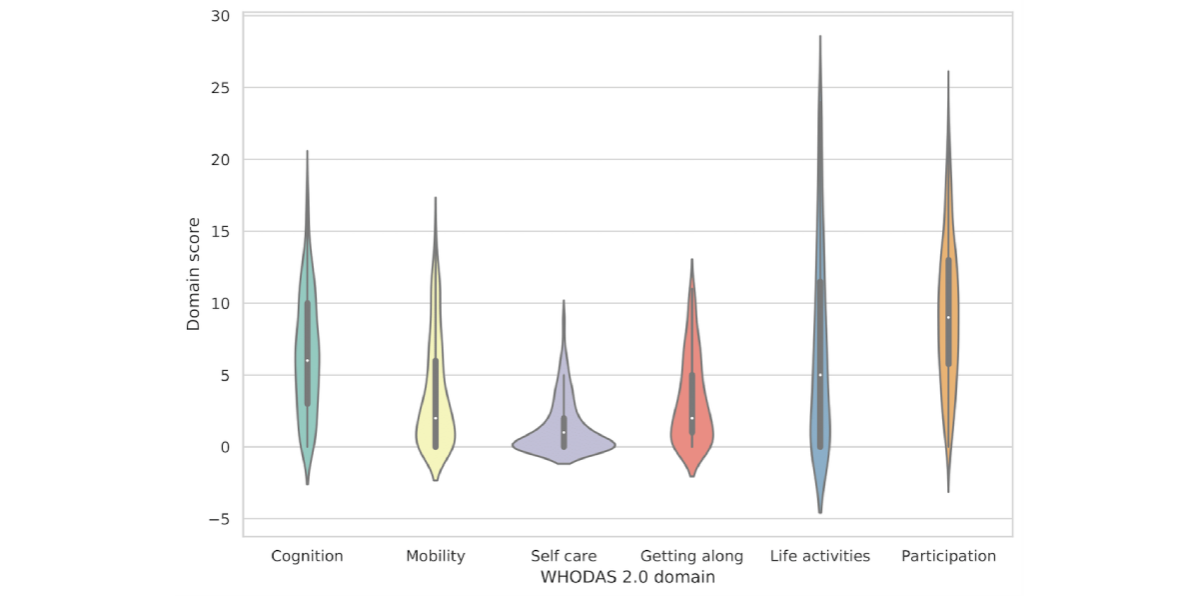
The distribution of World Health Organization Disability Assessment Schedule 2.0 functionality scores per domain in the patient cohort. WHODAS: World Health Organization Disability Assessment Schedule.

After our data filtering (Figure 1), 1526 WHODAS 2.0 domain entries of 396 participants collected between January 2017 and April 2021 were selected for our analysis. The cohort of patients had a median age of 44 (IQR 33-53) years at baseline, 63.13% (250/396) were female participants, and 29.04% (115/396) were male participants. Age and gender information were unavailable for 8.08% (32/396) and 7.83% (31/396) of the participants, respectively. Sociodemographic information was not included as input to the model. Figure 2 depicts an overview of the WHODAS 2.0 domain score distributions in the overall population in the form of violin plots, encompassing the summary statistics and the density of each domain score. The white dot in the middle is the median value, and the thick black bar in the center represents the IQR. Table S1 in Multimedia Appendix 1 provides the numerical statistics on the distributions.

### Results of Feature Selection and Predictive Modeling

We performed the feature selection using SFS, followed by the training of unregularized linear regressors with the best feature subset for each domain. We then compared the performance to linear regressors trained on the entire feature set. Table 1 shows the domain prediction errors as MAE and MAPE for both experimental set-ups. Note that these scores are inversely oriented, meaning that lower values indicate better performance.

**Figure 3:**
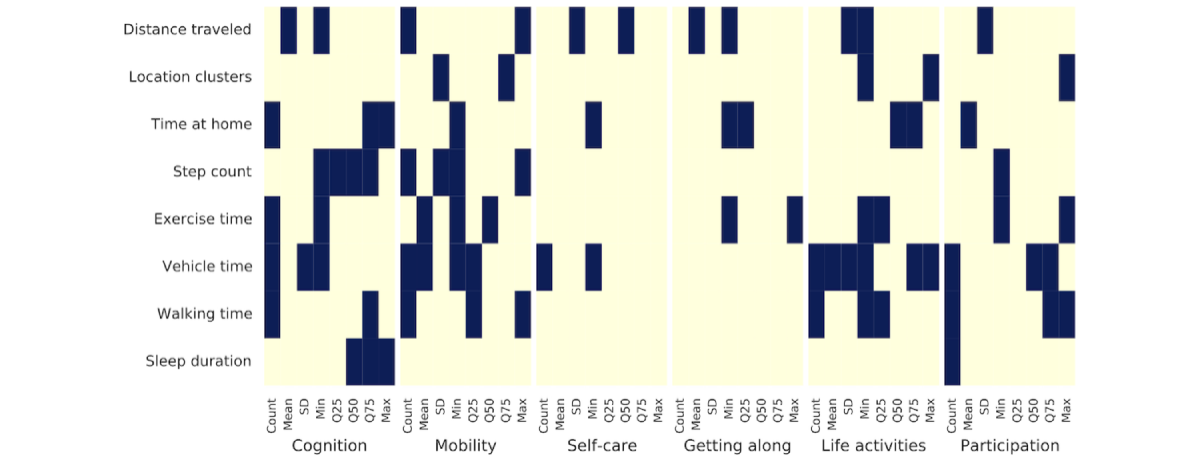
Feature selection results per World Health Organization Disability Assessment Schedule 2.0 domain.

**Figure 4:**
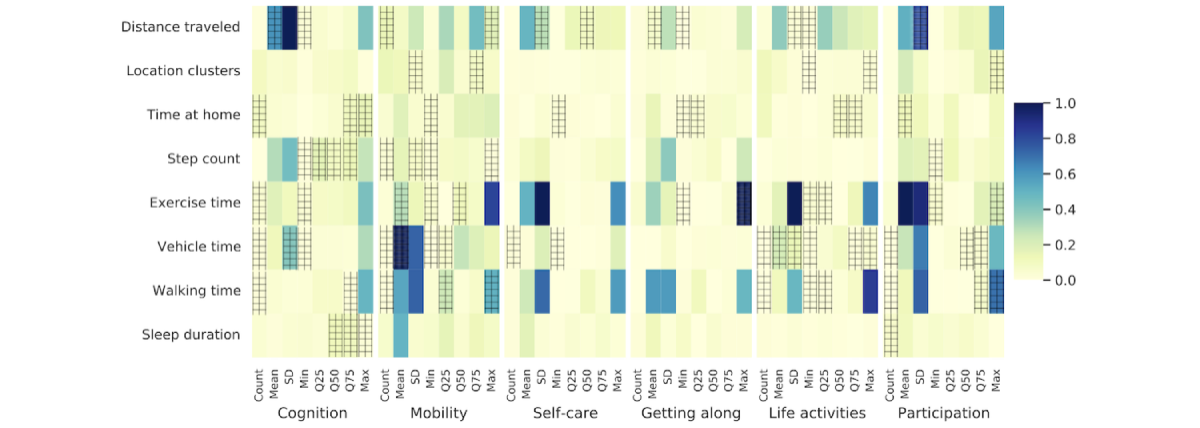
The feature importance of the linear regressor with all features per World Health Organization Disability Assessment Schedule 2.0 domain (shaded boxes) overlaid with the selected ones (hatched area). The shading indicates the size of the regression coefficient, while the hatching marks the selected feature using sequential forward selection.

Figure 3 summarizes the best feature subsets selected across all 6 domains (refer to Table S2 in Multimedia Appendix 1 for a detailed overview). In each case, the models consistently discarded a majority of input features, resulting in a reduction of the feature space to 19, 19, 5, 6, 17, and 13 features from 64 for the respective domains. Figure 4 illustrates the feature weights of the linear regressor trained with all features per domain, with the best subset features indicated by a grid overlay. To facilitate comparison, the absolute value of regression coefficients per domain model was normalized within the range of 0 to 1. While each feature statistic is not shared, it can be seen that the models coincide in the feature groups important to all feature models. Both models capture the relevant data from each feature group but use different statistics. Since the extracted statistics (count, mean, min, max, and quartiles) are interrelated, choosing a subset of these statistics can summarize the relevant information captured by the regressor.

Across all domains, at least 1 statistic related to distance traveled and time spent at home was consistently selected. These features impart information on daily movement patterns, which may indicate many elements of an individual’s lifestyle, including work (or lack of work), socialization (in and out of the home), and isolation, among many others. This focus on movement patterns was further reinforced by including vehicle time, step count, and walking time statistics across multiple domains. Step count and walking time are additional physical activity descriptors, along with exercise time. Physical activity biomarkers proved important in the cognition, mobility, life activities, and participation domains. Sleep-related biomarkers, however, were exclusively selected for the cognition and participation domains. Physical well-being in both exercise and sleep is reasonably related to these domains. The self-care domain and getting along domain were described with the lowest amount of important features compared to the 4 other domains, indicating a different pattern of feature relevance.

The performance results presented in Table 1 demonstrate that the regression models trained on the reduced feature space slightly outperformed those that were trained on all features. Regression models estimate parameters for each term in the model, and the presence of noninformative variables can introduce uncertainty, thereby diminishing the overall performance. However, the marginal difference observed suggests that both models can capture relevant information. Despite the model trained on all features being burdened with a larger number of features and lacking regularization, it still manages to retain some level of effectiveness in regression.

**Table 1:** Regression evaluation metrics over the 4 folds.

WHODAS^a^ 2.0 domain	Score range	Predicting with all 80 features		Predicting with selected features		
		MAE^b^, mean (SD)	MAPE^c^ (%), mean (SD)	Features selected, n	MAE, mean (SD)	MAPE (%), mean (SD)
Cognition	0-20	3.76 (3.21)	18.84 (16.09)	19	3.55 (2.90)	17.76 (14.54)
Mobility	0-16	3.50 (2.44)	21.91 (15.26)	19	3.40 (1.98)	21.26 (12.42)
Self-care	0-10	1.56 (1.75)	15.69 (17.54)	5	1.48 (1.50)	14.86 (15.09)
Getting along	0-12	3.13 (2.72)	26.11 (22.74)	6	2.44 (1.79)	20.37 (14.96)
Life activities	0-24	7.57 (6.40)	31.56 (26.66)	17	6.53 (5.40)	27.21 (22.52)
Participation	0-24	3.88 (3.14)	16.16 (13.10)	13	3.73 (2.69)	15.54 (11.23)

^a^WHODAS: World Health Organization Disability Assessment Schedule.

^b^MAE: mean absolute error.

^c^MAPE: mean absolute percentage error.

## Discussion

### Principal Findings

In this study, our objective was to predict WHODAS 2.0 functionality scores for each domain using only passively collected digital biomarkers. To achieve this, we used statistical feature engineering techniques, followed by a simple machine learning approach that involved selecting features through SFS for linear regression. This approach demonstrated the potential to predict functionality from passively sensed data. Additionally, by using a straightforward linear regression model, we ensured the interpretability of the model’s decisions.

Extracting statistical measures of the time-series sequences allowed dealing with missing data without applying imputation techniques; however, certain entries had to be excluded due to their limited information content (eg, sequences with completely missing observations per feature). Our focus was to identify the most relevant features from an ample feature space and eliminate noninformative or redundant predictors from the model for each domain. We found that 5-19 features were sufficient for each domain, and the most relevant being the distance traveled, time spent at home, time spent walking, exercise time, and vehicle time. These features are biomarkers for daily movement patterns and individuals’ physical activity that were most informative for linear regression.

The models using feature selection outperformed those using the entire feature space across all domains. They achieved accurate predictions of patients’ WHODAS 2.0 functionality scores per domain, with a maximum MAPE of 27.21% on the life activities domain and a minimum MAPE of 14.86% on the self-care domain. These errors are reasonable for a linear regression performing a complicated ordinal classification task.

The overall model performance can be explained by the distribution of the outcome variable within the respective data sets: mid-range values dominate in each domain; therefore, it becomes harder for the models to predict the more extreme scores. This issue is exacerbated by the fact that the target variable’s distribution is skewed rather than normally distributed, potentially affecting the performance of a regression model. Additionally, both the presence of missing values and a target variable that is better suited for ordinal classification presented challenges that impacted performance.

It is worth noting that the domains with the fewest selected features and the smallest range of possible scores exhibited the lowest and highest MAPE values, respectively. Feature selection did not alter the error ranking of the domains when compared to the model that used all features.

Regarding feasibility, an average MAPE (across the 6 domains) of 19.5% was a reasonable error for a simple yet interpretable model. WHODAS 2.0 domain or total scores cut-offs tend to be used clinically based on population norms [14]. A total score of 50, which is similar to a percentile score in how it is rescaled from 0 to 100, almost represents the 95th population percentile [14]. Our model could be feasibly used to determine cases of severe disability even with an error margin of around 20%. As mentioned previously, the most important use case would be passive continuous assessment of patients. With the current level of error, using this simple yet effective model would be feasible for detecting moderate or severe changes in functionality. Additionally, the experiments presented herein do not take into account the continuous streaming of data that can further help the model to be more accurate globally and for a specific user. We believe that the framework and approach presented, especially with room to improve, show promising results for creating a continuous assessment and monitoring system that could alert physicians to patient loss of functionality and allow for just-in-time interventions.

### Limitations

Although our approach showed promising results, it also has limitations. The automatically generated wearable device data were passively collected in a real-world setting. This is a strength in ecological validity; however, it also introduces challenges, such as significant missing data and noise, which are commonly encountered in real-world passive data acquisition. Moreover, there are also data quality problems: missing data due to users not wearing the device or incorrect data arising from the device malfunctioning. These issues may have adversely affected the predictive performance of our model and biased the importance of certain variables. Missing data also posed a problem in the features used from the eB2 database, as other helpful features, such as app usage and phone unlocks, which are directly related to social domains, were filtered out, causing a severely reduced data set. Consequently, this led to a substantial reduction in our data set. The data set presents many challenges because it is the result of merging 2 real-world databases with missing values, erroneous entries, and noise.

Another limitation arises from the fact that, in many cases, individuals have only a single score, which does not allow for training personalized models that could better account for the intraindividual variations. While step data can provide relevant information for determining mobility, factors such as an individual’s lifestyle, work conditions, and other variables greatly influence step count. We hypothesize that a model that learns individual variability and patterns and then examines the population would better suit this task. However, obtaining longitudinal large population data sets that combine clinical and wearable data poses considerable challenges.

### Conclusions

Our work is the first to suggest a machine learning–based approach for assessing WHODAS 2.0 functionality from passively sensed data. The results demonstrate the feasibility of designing a pipeline that passively monitors patients’ functionality over time. However, it is important to note that predicting scores for each WHODAS 2.0 domain equally well poses a significant challenge. Nevertheless, the feature selection approach provides insight into relevant behavioral measures contributing to improved prediction performance. This aspect of our approach enhances the interpretability of the results, which is important for real-world and clinical applications.

Moving forward, an interesting avenue to explore is deep learning-based temporal methods that operate on the data at a more refined time scale. Instead of relying on statistical feature engineering and selection, these models can learn representations directly from raw input sequences that are most relevant to the prediction problem. We will further investigate the possibilities of specialized models for individual patients to understand the functionality evolution better, improve prediction accuracy, and provide interpretable model outputs to clinicians. This line of investigation holds promise for advancing the field and addressing the challenges associated with predicting WHODAS 2.0 functionality using passive data collection methods.

## Appendix

Multimedia Appendix 1 Tables of the WHODAS 2.0 functionality scores per domain in the patient cohort and the lists of selected features per domain after sequential feature selection.

## Abbreviations

EMA: ecological momentary assessment
MAE: mean absolute error
MAPE: mean absolute percentage error
PREM: patient-reported experience measure
PROM: patient-reported outcome measures
SFS: sequential forward selection
WHO: World Health Organization
WHODAS: World Health Organization Disability Assessment Schedule

## Data Availability

The data sets generated and analyzed during this study are not publicly available due to their personally identifiable and sensitive health information content but are available from the corresponding author on reasonable request.
