# Supplementary material for: Continuous Assessment of Function and Disability via Mobile Sensing: Real-World Data-Driven Feasibility Study
Source: JMIR Form Res. 2023 Oct 30;7:e47167. doi: 10.2196/47167 (PMC10644188; doi:10.2196/47167)
Supplement: Multimedia Appendix 1 [file formative_v7i1e47167_app1.pdf]

Multimedia Appendix 1

Table S1. The distribution of WHODAS 2.0 functionality scores per domain in the patient cohort.

WHODAS domain	Number of patients	Number of entries	Score range	Overall mean (SD)	Overall median (IQR)
Cognition	309	323	0-20	6.41 (4.13)	6 (3, 10)
Mobility	370	383	0-16	3.83 (3.84)	2 (0, 6)
Self-care	341	353	0-10	1.39 (1.91)	1 (0, 2)
Getting along	218	225	0-12	3.28 (3.03)	2 (1, 5)
Life activities	227	235	0-24	6.94 (6.84)	5 (0, 11)
Participation	320	332	0-24	9.20 (5.01)	9 (5, 13)

Table S2. Lists of selected features per domain after applying sequential feature selection. Notations: SD = standard deviation, Q25/50/75 = 25th, 50th, 75th quantile.

WHODAS domain	Number of features selected	Selected features
**Cognition**	19	Mean distance traveled
		Minimum distance traveled
		Number of time spent at home entries
		Q75 of time spent at home
		Maximum time spent at home
		Minimum step count
		Q25 of step count
		Q50 of step count
		Q75 of step count
		Number of exercise time entries
		Minimum time spent exercising
		Number of vehicle time entries
		SD of vehicle time
		Minimum vehicle time
		Number of walking time entries
		Q75 of time spent walking
		Q50 of sleep duration
		Q75 of sleep duration
		Maximum sleep duration
**Mobility**	19	Number of distance traveled entries
		Maximum distance traveled
		SD of number of visited locations
		Q75 of number of visited locations
		Minimum time spent at home

		Number of step count entries<br>SD of step count<br>Minimum step count<br>Maximum step count<br>Mean time spent exercising<br>Minimum time spent exercising<br>Q50 of time spent exercising<br>Number of vehicle time entries<br>Mean vehicle time<br>Minimum vehicle time<br>Q25 of vehicle time<br>Number of walking time entries<br>Q25 of time spent walking<br>Maximum time spent walking
**Self-care**	5	SD of distance traveled<br>Q50 of distance traveled<br>Minimum time spent at home<br>Number of vehicle time entries<br>Minimum vehicle time
**Getting along**	6	Mean distance traveled<br>Minimum distance traveled<br>Minimum time spent at home<br>Q25 of time spent at home<br>Minimum time spent exercising<br>Maximum time spent exercising
**Life activities**	17	SD of distance traveled<br>Minimum distance traveled<br>Minimum number of visited locations<br>Maximum number of visited locations<br>Q50 of time spent at home<br>Q75 of time spent at home<br>Minimum time spent exercising<br>Q25 of time spent exercising<br>Number of vehicle time entries<br>Mean vehicle time<br>SD of vehicle time<br>Minimum vehicle time<br>Q75 of vehicle time<br>Maximum vehicle time<br>Number of walking time entries<br>Minimum time spent walking<br>Q25 of time spent walking

**Participation**	13	SD of distance traveled<br>Maximum number of visited locations<br>Mean time spent at home<br>Minimum step count<br>Minimum time spent exercising<br>Maximum time spent exercising<br>Number of vehicle time entries<br>Q50 of vehicle time<br>Q75 of vehicle time<br>Number of walking time entries<br>Q75 of walking time<br>Maximum time spent walking<br>Number of sleep duration entries
